# Seroprevalence and risk factors of *Toxoplasma gondii* in sheep and goats of North West Province, South Africa

**DOI:** 10.1186/s12917-024-03939-7

**Published:** 2024-03-26

**Authors:** Mthokozisi Masombuka, Malekoba B.N. Mphuthi, Yusuf B. Ngoshe, Gloria Mokolopi, Nomakorinte Gcebe

**Affiliations:** 1 Department of Agriculture and Animal Health, College of Agriculture and Environmental Sciences, University of South Africa, Science Campus, Private Bag X6, Florida, Johannesburg, 1710 South Africa; 2 Department of Animal Health, School of Agricultural Sciences, North-West University, Private Bag X2046, Mmabatho, 2735 South Africa; 3 Epidemiology Section, Department of Production Animal Studies, Faculty of Veterinary Science, University of Pretoria, Private Bag X04, Onderstepoort, Pretoria South Africa; 4 Agricultural Research Council– Onderstepoort Veterinary Research, Bacteriology, and Zoonotic Diseases Laboratory, 100 Old Soutpan Road, Onderstepoort, Pretoria 0110 South Africa

**Keywords:** *Toxoplasma gondii*, Sheep, Goats, North West Province, South Africa, Risk factors, ELISA

## Abstract

**Background:**

The protozoan parasite *Toxoplasma gondii* causes toxoplasmosis, one of the most prevalent parasitic zoonotic diseases with significant economic and public health implications worldwide. Infection with the parasite has a significant adverse effect on sheep and goat production and can frequently go undetected in the herd, resulting in abortions and weak or dead offspring. Although there are few studies on seroprevalence and risk factors associated with *T. gondii* infections in livestock in other provinces of South Africa, there is no data in the North West province. Therefore, a cross-sectional study was conducted to investigate the seroprevalence of *T. gondii* and risk factors associated with exposure in sheep and goats of the North West province of South Africa. Sera from 439 livestock (164 sheep and 285 goats) were collected and analysed for the presence of *T. gondii* IgG antibodies using indirect ELISA (Enzyme-linked immunosorbent assay). An assessment of potential risk factors in farms associated with seropositivity was also conducted using a structured questionnaire.

**Results:**

Out of the 439 tested sheep and goats, 13.9% (61/439) were positive for IgG antibodies against *T. gondii*. Sheep and goats had seroprevalences of 19.5% (32/164) and 10.5% (29/275) respectively. In the multivariable logistic regression model, the risk of acquiring *T. gondii* was significantly higher in the mixed breed [Odds ratio (OR) = 71.07; 95% confidence interval (CI): 266.8-1893.1; *p* < 0.011)] animals than white dorper sheep and in farms that burn or bury aborted material (OR = 42.04; CI: 179.9-982.5; *p* = 0.020) compared to those that only burn aborted material. The risk was lower for the farms in Kagisano-Molopo (OR = 0.00; CI: 0.0-25.4; *p* = 0.015) and Mahikeng (OR = 0.00; CI: 0.0-4.9; *p* < 0.001) local municipalities than Greater Taung local municipality, and for the animals that drink water from dams (OR = 0.03; CI: 0.2–58.8; *p* = 0.021) than those that drink from boreholes.

**Conclusion:**

The seroprevalence and risk factors associated with transmission observed show that *T. gondii* infection is widespread in sheep and goats of the North West province.

**Supplementary Information:**

The online version contains supplementary material available at 10.1186/s12917-024-03939-7.

## Background

*Toxoplasma gondii (T. gondii)*, is a ubiquitous obligate intracellular protozoan parasite that causes toxoplasmosis in warm-blooded animals, including humans. The parasite is the only species of the Toxoplasma genus and is a member of the Apicomplex phylum, Conoidasida class, Eucoccidiorida order, and Sarcocystidae family [1]. Although many animal species serve as intermediate hosts, only domestic cats, and other felids are definitive hosts and the only species that shed *T. gondii* oocysts into the environment which makes them important in the epidemiology of the parasite [1–3]. Both animals and humans can become infected through consumption of food sources containing tissue cysts or contaminated with oocysts [4]. Even though infection with *T. gondii* may show no clinical symptoms, in some intermediate hosts, toxoplasmosis may result in huge economic losses due to reproductive disorders such as abortions and stillbirths [3, 5, 6]. In sheep and goats, toxoplasmosis manifests as a disease of pregnancy, resulting in foetal resorption, stillbirth, mummified lambs, and neonatal mortality [7–9].

Although the disease is mostly asymptomatic or only show mild clinical signs in healthy individuals, it may result in miscarriages, stillbirths, and even death in individuals with compromised immune systems, such as those with human immunodeficiency virus (HIV) [10, 11]. Toxoplasmosis is prevalent in both animals and humans worldwide, including South Africa, and results in huge economic losses in sheep and goats and has serious public health implications which results in congenital defects and ocular diseases in humans [12–16]. According to a 2017 study conducted in South Australia by Ryan O’Handley, the cost of sheep toxoplasmosis was estimated to be 70 million Australian dollars, as per a recent ABC news report (https://www.abc.net.au/news/rural/2017-02-07/toxoplasmosis-costs-south-australian-sheep-producers/8245676, accessed on 10/01/2024). In another study conducted by Bennett and Ijpelaar in Britain in 2005, they estimated the annual number of affected sheep to be about 334 000, with disease effects of 9.1 million pounds because of abortion or stillbirth and 3.2 million pounds for disease control [17]. Despite this, it is still one of the understudied zoonoses with few reports on seroprevalence and risk factors associated with transmission in livestock in South Africa.

A seroprevalence of 6.0%, 2.7%, 6.3%, and 8% in sheep of Gauteng, Free State, KwaZulu-Natal, and Western Cape, respectively was reported in the year 2007 [18, 19]. In the year 2020, a seroprevalence of 15.2% was also reported in cattle of the Mnisi community in Mpumalanga province [4], and 20.8% in cattle from the high throughput Klerksdorp and Rustenburg abattoirs of the North West province [20]. A more recent study conducted in the Eastern Cape province reported a higher seroprevalence in sheep at 64.46%, followed by goats at 53.91%, pigs at 33.90%, chickens at 33.58%, and cats at 32.11% [13].

A study on risk factors associated with *T. gondii* infection in domestic animals by Tagwireyi [13] found, age, animal production system, cat faeces and feed disposal, climate, location, rodent control, and seropositive cats to be risk factors associated with increased seropositivity of *T. gondii* for sheep, goats, chicken, and pigs in the Eastern Cape. It is important to control *T. gondii* infection in sheep and goats not only for reproduction purposes but also for public health reasons. In this current study, we report seroprevalence and risk factors associated with *T. gondii* exposure in sheep and goats in the North West province of South Africa.

## Materials and methods

### Study area

**Fig. 1:**
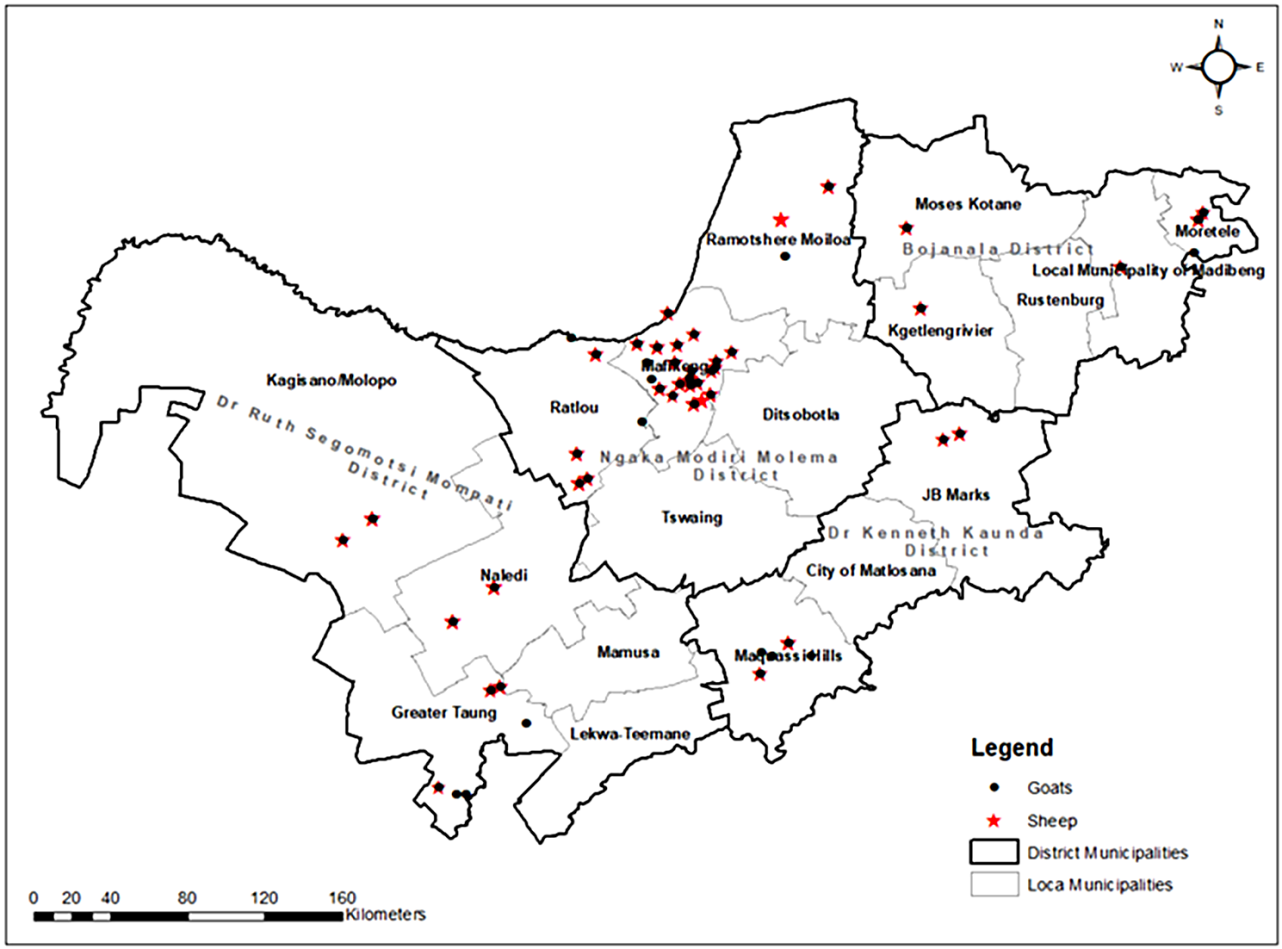
Map of North West province districts and local municipalities where sampling was conducted

A cross-sectional study was conducted in the North West province, which is in the northern part of South Africa near the Botswana border and bordered by the Free State to the south, the East Rand to the east, and the Kalahari Desert to the west. It has a population of 3,748 436 people and a 104 882 km² area. Most of the province is made up of flat, sparsely vegetated plains. The province is organized into 18 local municipalities from its four district municipalities. Samples were collected in all four districts (Fig. 1). Villages were selected randomly from each local municipality. In each village, efforts were made to consider all directions (East, West, North, and South of the village using an abstract transect) to avoid bias. A convenient sampling was used to select farmers, and only farmers who gave consent to participate in the study were included. On the farm level, only healthy adult animals and female animals with a history of abortion as pointed out by the farmer were conveniently included in the study.

### Sample size and study population

To estimate the sample size for this study, a formula by [21]: $n=\left[\right(1.96)^2 Pexp (1-Pexp\left)\right]/d^2$ [21] was used, where p is the estimated prevalence, d the estimated precision, and *n* the estimated sample size. Due to the lack of data on *T. gondii* prevalence in the study population, an expected prevalence (P exp) of 50% was used and 5% was set as the estimated precision (d), giving a required minimum sample size of 384. However, for the study, 439 (164 sheep and 275 goats) animals were sampled.

### Questionnaire survey

A structured questionnaire was used to determine the risk factors that play role in the exposure of the animals to *T. gondii* infections. The information collected from questionnaire included age, breed, sex, the origin of the animals (local market, local market and auction, local market and own breed, own breed), location of the farms (district municipality, local municipality), type of farm (communal, commercial), type of breeding (natural, artificial insemination), history of abortion, disposal of aborted material (dispose of, dispose in a hole and close, do not dispose of, used as fertilizer for plants), type of feeding (free grazing, free grazing and farm fed, free grazing and home fed, home fed), feed storage (not applicable, stored in a car garage, stored in a designated room, stored in a designated shack), disposal of manure (dispose of, dispose of in a hole and close, do not dispose of, use as fertilizer for plants), water source (borehole, borehole and dam, borehole and tap water, borehole and river, dam, tap water) and presence of cats in the farms.

### Sample collection

Venous blood was collected using BD-Vacutainer® SST™ II Advance 10 ml serum collection tubes. The blood samples were kept in a cooler with ice packs and transported to the laboratory. Sera was harvested the same day by centrifuging the clotted blood at 1000 x *g* for 10 min and transferred into collection tubes. Sera was stored at -20 °C until analysis.

### Serological assay

The sera were used to detect the *T. gondii* IgG antibodies using the IDEXX *T. gondii* 2/strip antibody test kit following the manufacturer’s instructions (IDDEX Laboratories, Liebelfld-Bern, Switzerland). Frozen sera and reagents from the test kit were brought to room temperature and concentrated wash buffer was diluted 10 times with distilled water. The diluted buffer was used to dilute the positive, and negative controls, as well sera 400-fold. A volume of 100 µl of the diluted controls and sera was transferred into the *T. gondii* antigen-coated plate wells and incubated for an hour at 37 °C. The plate was washed three times with 300 µl of the diluted wash buffer after incubation. The wash buffer residues were removed by gently tapping the plate on absorbent paper. A 100 µl of the conjugate was added to each well and incubated for an hour at 37 °C. The plates were again washed three times with 300 µl wash buffer with wash buffer residues removed as described above, and 100 µl of 3, 30, 5, 50-Tetramethylbenzidine (TMB) was added and incubated for 15 min in the dark at room temperature. The stop solution of 100 µl to was then added to each well following incubation. The absorbance at 450 nanometres was immediately measured using a Thermo Labsystems MultiskanMS Original microplate reader (Thermo Fischer Scientific, Waltham, MA, USA).

The assay validity was evaluated using the following criteria: The two negative controls average (NCx) optical density value at 450 nm (A450) should be less than or equal to (≥) 0.500 to determine the validity of the test. At 450 nm, the two positive controls average value (PCx) should be ≤ 2.500 and the PCx-NCx should be larger than or equal to (≤) 0.300. The following formula was used to determine sample to positive (S/P) ratio:

$${\text{S/P}}\% = 100\frac{{{\text{Sample}}\left( {{\text{A}}450} \right) - {\text{NCx}}\left( {{\text{A}}450} \right)}}{{{\text{PCx}}\left( {{\text{A}}450} \right) - {\text{NCx}}\left( {{\text{A}}450} \right)}}$$

For results interpretation, S/P % <20 represented a negative result, 20 ≤ S/P % <30 represented a suspect, 30 ≤ S/P% <100 represented a weak positive result, and S/P % ≥100 represented a positive result.

### Data analysis

The data was captured and cleaned using a Microsoft Excel spreadsheet and analysed using Stata 14.0 (StataCorp, College Station, Texas, USA). To determine the association between the potential risk factors and seropositivity of *T. gondii*, a univariate analysis using a 2 × 2 contingency table was done and a Chi-square test with appropriate p-value was reported. Chi-square test *P* ≤ 0.2 was used as an inclusion criterion in the multiple logistic regression model as recommended by [22]. Multivariable analysis was done using multilevel logistic regression models and a stepwise procedure was performed by removing variables with *P* > 0.05. The Akaike’s second-order information criterion (AIC) was calculated for each model, and the process was repeated until the model with the lowest AIC was identified. Results for the odds ratios with a 95% confidence interval were reported.

## Results

Of the 439 sheep and goats tested sera, 13.9% (61/439) were positive for IgG antibodies against *T. gondii*. The seroprevalence of *T. gondii* in sheep and goats was 19.5% (32/164) and 10.5% (29/285) respectively. The seroprevalence was higher in females than in males at 14.6% (59/405) and 5.9% (2/34), respectively (Table 1). In the univariate analyses, significant association with *T. gondii* seropositivity was seen for age (*P* = 0.170), sex (*P* = 0.160), species (*P* = 0.009), breed (*P* = 0.028), district municipality (*P <* 0.001), local municipality (*P* < 0.001), the origin of animal (*P <* 0.001), source of water (*P* = 0.067), history of abortion (*P* = 0.005), disposal of aborted material (*P* = 0.014), disposal of manure (*P-*= 0.027), feeding system (*P* = < 0.001), and presence of cats in the farms (*P <* 0.001) (Table 1). There was no significant association with *T. gondii* seropositivity in breeding (*P* = 0.330), type of farm (*P* = 0.372), and feed storage (*P* = 0.309) (Table 1).

**Table 1:** Univariate risk analysis of factors associated with *T. gondii* seropositivity in sheep and goats

Risk factor level	Number of sampled animals	Number testing positive	Percentage seropositivity	95% CI	*p*-Value
**Age**					
1–2	145	17	11.7	28.8–37.6	0.170
> 2–3	223	33	14.8	46.1–55.5	
> 3–4	55	11	20.0	9.7–16.0	
> 5	16	0	0	2.2–5.9	
**Sex**					
Female	405	59	14.6	89.3–9.4	0.160
Male	34	2	5.9	5.6–10.7	
**Species**					
Goat	275	29	10.5	58.0-67.1	0.009
Sheep	164	32	19.5	32.9–42.0	
**Breed**					
Boerbok	108	7	6.5	20.8–28.9	0.028
Dorper	18	0	0	2.6–6.4	
Kalahari red	1	0	0	0.3–1.6	
Mixed	291	53	18.2	61.7–70.6	
Polled Dorset	2	0	0	0.1–1.8	
Saanen	2	0	0	0.1–1.8	
White Dorper	17	1	5.9	2.4–6.2	
**Type of farm**					
Commercial	83	9	10.8	15.5–22.9	0.372
Communal	356	52	14.6	77.1–84.5	
**District municipality**					
Bojanala Platinum	77	4	5.2	14.3–21.4	< 0.001
Dr Kennet Kaunda	30	1	3.3	4.8–9.6	
Dr Ruth Segomotsi Mompati	87	19	21.8	16.3–23.8	
Ngaka Modiri Molema	245	37	15.1	51.1–60.4	
**Local Municipality**					
Greater Taung	31	1	3.2	5.0-9.9	*<* 0.001
JB Marks	12	0	0	1.6–4.8	
Kagisano-Molopo	31	5	16.1	5.0-9.9	
Kgetlengrivier	10	0	0	1.2–4.2	
Madibeng	6	0	0	0.6- 3.0	
Mahikeng	165	7	4.2	33.2–42.2	
Maquassi Hills	18	1	5.6	2.6–6.4	
Moretele	41	3	7.3	6.9–12.5	
Moses Kotane	20	1	5.0	2.9–6.9	
Naledi	25	13	52.0	3.8–8.3	
Ramotshere Moiloa	22	9	40.9	3.3–7.5	
Ratlou	58	21	36.2	10.3–16.7	
**Presence of cats**					
Yes	129	31	24.0	25.3–33.8	*<* 0.001
No	310	30	9.7	66.2–74.7	
**Breeding**					
Natural	422	60	14.2	93.9–97.6	0.330
Natural and AI	17	1	5.9	2.4–6.2	
**History of abortion**					
Aborted	80	19	23.8	14.9–22.1	0.005
No history	359	42	11.7	77.9–85.1	
**Disposal of aborted material**					
Burn	8	0	0	0.9–3.6	0.014
Burn or bury	9	3	33.3	1.1–3.9	
Bury	90	15	16.7	16.9–24.6	
Burn or hang on the tree	64	11	17.2	11.6–18.2	
No history of abortion	251	32	12.7	52.5–61.8	
Send to the state vet	17	0	0	2.4–6.2	
**Disposal of manure**					
Dispose	59	8	13.6	10.6–16.9	0.027
Dispose in a hole and close	10	0	0	1.2–4.2	
Do not dispose	71	3	4.2	13.0-19.9	
Use as fertilizer for plants	299	50	16.7	63.6–72.3	
**Origin of animals**					
Auction	112	7	6.3	21.6–29.8	< 0.001
Local market	174	28	16.1	35.2–44.3	
Local market and auction	19	12	63.2	2.8–6.7	
Local market and own breed	30	8	26.7	4.8–9.6	
Own breed	104	6	5.8	19.9–27.9	
**Source of water**					
Borehole	261	41	15.7	54.8–63.9	0.067
Borehole and dam	76	9	11.8	14.0-21.6	
Borehole and tap water	26	0	0	4.1–8.6	
Borehole and river	21	1	4.8	2.6–6.4	
Dam	37	4	10.8	6.2–11.4	
Tap water	21	6	28.6	3.1–7.2	
**Feeding system**					
Free grazing	101	17	16.8	19.3–27.2	*<* 0.001
Free grazing and farm-fed	130	3	2.3	25.5–34.1	
Free grazing and home-fed	199	41	20.6	40.7–50.0	
Home fed	9	0	0	1.1–3.9	
**Feed storage**					
Not applicable	91	17	18.7	17.2–24.8	0.309
Stored in a car garage	28	5	18	4.4–9.1	
Stored in a designated room	302	38	12.6	64.3–72.9	
Stored in a designated shack	18	1	5.6	2.6–6.4	

CI: Confidence interval.

In the final multivariable logistic regression model, only breed, district municipality, local municipality, disposal of aborted material, and water source, remained as significant risk factors (Table 2). The risk of acquiring *T gondii* was higher in the mixed breed (OR = 71.07; 95% CI: 266.81-1893.128; *p* < 0.011) animals compared to white dorper and in farms that burn or bury aborted material (OR = 42.04; CI: 179.91-982.49; *p* = 0.020) compared to those that burn. Animals that drink water from dams had a lower risk (OR = 0.03; CI: 0.15–58.84; *p* = 0.021) than those that drink from boreholes. The risk was lower for farms located in Kagisano-Molopo (OR = 0.00; CI: 3.46e − 6-25.41; *p* = 0.015) and Mahikeng (OR = 0.00; 3.39e − 3-4.94; *p* < 0.001) local municipalities than Greater Taung local municipality.

**Table 2:** Multivariate logistic regression analysis of risk factors associated with *T. gondii* seropositivity in sheep and goats

Risk factor	Odds Ratio	95% CI	*p*-Value
**Age**			
1–2*			
> 2–3	1.0	35.-285.14	0.989
> 3–4	1.3	35.4-510.5	0.664
> 5			
**Species**			
Goat*			
Sheep	1.4	53.9-364.1	0.489
**Breed**			
Boerbok*			
Dorper			
Kalahari red			
Mixed	71.1	266.8-1893.1	0.011
Polled Dorset			
Saanen			
White Dorper	0.8	3.2-1959.2	0.887
**District**			
Bojanala Platinum*			
Dr Kennet Kaunda	0.1	0.1-1096.2	0.362
Dr Ruth Segomotsi Mompati	2.6	3.5-188.3	0.666
Ngaka Modiri Molema	0.1	0.0-745.9	0.247
**Municipality**			
Greater Taung*			
JB Marks			
Kagisano-Molopo	0.00	0.0-25.4	0.015
Mahikeng	0.00	0.00-4.9	< 0.001
Maquassi Hills			
Moretele	0.4	0.8- 2424.7	0.691
Moses Kotane			
Naledi	0.00	0.00- 133.8	0.065
Ramotshere Moiloa	0.1	2.3- 1284.9	0.709
Ratlou			
**Presence of cats**			
Yes	1.8	35.4- 910.5	0.480
No*			
**History of abortion**			
Aborted			
No history*	0.2	26.4-206.6	0.191
**Disposal of aborted material**			
Burn*			
Burn or bury	42.0	179.9-982.5	0.020
Bury	1.7	36.5-746.7	0.515
Burn or hang on the tree	0.8	3.8-1875.6	0.914
No history of abortion			
Send to the state vet			
**Animal origin**			
Auction*			
Local market	1.6	22.5- 1369.1	0.592
Local market and auction	14.9	85.3-25969.5	0.064
Local market and own breed	0.9	3.7- 2362.3	0.967
Own breed	11.1	56.4-22058.7	0.113
**Source of water**			
Borehole*			
Borehole and dam	0.1	1.3-166.6	0.121
Borehole and tap water			
Borehole and river	28.8	49.2-168124.1	0.106
Dam	0.0	0.2–58.8	0.021
Tap water	0.3	2.5-285.5	0.275
**Feeding system**			
Free grazing*			
Free grazing and farm-fed	8.6	46.9- 15749.5	0.147
Free grazing and home-fed	0.8	14.6-474.2	0.836
Home fed			

CI: Confidence interval. *: Reference category.

## Discussion

For the first time in the North West province of South Africa, we determined the seroprevalence of *T. gondii* in sheep and goats, using an indirect ELISA. Overall, we found a seroprevalence of 13.9%, with sheep (19.5%) having a higher seroprevalence than goats (10.5%). The difference in seroprevalence between the two species could be attributed to the fact that goats are natural browsers that eat leaves and twigs from taller bushes and shrubs, making them less likely to ingest oocysts, whereas sheep are grazers who eat short grasses and clovers that are near the soil [23–25]. The overall seroprevalence reported in this study is lower than the 67.2% reported in Zimbabwe for sheep and goats [26], and the 64.5% and 53.9% for sheep and goats respectively, reported in the Eastern Cape in the year 2019 [13]. This pronounced higher differences could be attributed to the climatic variations between North West and Eastern Cape province (dry and semidesert vs. moist and humid), since the moist and humid environment is conducive for the survival of oocytes and spread compared to dry and semi-desert environment, leading to increased seropositivity [27, 28]. Seropositivity for goats agreed with the 10% found in Botswana [29]. In our study, females had seropositivity of 14.6% against 5.9% in males, a finding similar to the one from the Eastern Cape [13]. The difference in seropositivity observed between females and males could be due to hormonal fluctuations, immunosuppression associated with pregnancy, lactation stress, and physiological changes which makes females more susceptible to *T. gondii* infections than males [30]. A significant association between *T. gondii* seropositivity and breed was observed with mixed breed having the highest seropositivity (18.2%) when compared to other breeds. Inbreeding is known to weaken the genetic makeup of animals, thus making them more prone to diseases than pure breeds, which could have been the case with the higher seropositivity observed in this study [3, 31, 32]. The difference in seropositivity between the animals that were allowed to breed naturally (14.2%) and those that were artificially inseminated (5.9%) is consistent with previous studies on sexual transmission of the parasite using both the methods [3, 31, 33, 34]. This might indicate a possible sexual transmission through the natural breeding rather than contamination from non-sterile artificial insemination apparatus and procedures.

The presence of cats on the farms was a significant risk factor in this study, which was expected given their role as definitive hosts in the spread of *T. gondii* [3]. This finding was consistent with those that were reported in Romania [35]. Differences in seroprevalence among the district and local municipalities played a role in the exposure of the animals to the parasite, a result consistent with that of Eastern Cape, South Africa [13]. The different temperatures, humidity, and annual rainfall among the district municipalities could have contributed to the variation of seropositivity as the humidity in the Kagisano-Molopo local municipality and the hot weather in Mahikeng local municipality. These climatic variances agree with the ones found in the different regional states in Central Ethiopia where altitudes, rainfall and temperatures were different among the studied districts [36]. Free grazing and home fed animals system had a significant association with higher seropositivity compared to the rest of the feeding systems. These findings further support the role cats play in the epidemiology of the parasite as they could have potentially contaminated the feed of the animals [37–39]. Disposal of manure was also a significant factor with the highest seropositivity observed from farms that uses manure as a fertilizer. This significance is no surprise particularly when factoring in the presence of cats in the farms, an indication of shedding oocysts on the feed, soil, pastures, manure, and water sources of the animals which increases their risk of infection [16, 40].

The introduction of diseases that have not previously existed or have been eradicated in a herd is highly influenced by the origin of newly introduced animals. This was the case even with the significant finding in this study, which demonstrated seropositivity rates of 62.2% on farms that purchase animals from local markets and auctions, compared to those only originating from auctions, indicating a potential for exposure as reported in the Eastern Cape province [13]. The animals with a history of abortion were more susceptible to exposure than the ones that did not have (23.8% vs. 11.7%). A study in Botswana reported similar findings from animals with a history of abortion [29]. Animals from farms that burn or burry aborted material had the highest seropositivity of 33.3% than animals from farms that send aborted material to sate vet (0%), which demonstrates the importance of proper disposal of aborted material in preventing the spread of *T. gondii* as pets can get infected by eating the aborted material containing *T. gondii* tissue cysts or tachyzoites [41, 42]. This significance corroborates previous findings where high seropositivity was found in farms that bury or feed the aborted material to pets [43, 44].

According to the final multivariable logistic model, mixed-breed Dorpers had a higher risk of acquiring an infection than white Dorpers, which is most likely because of the subpar hygienic-sanitary practices used to maintain mixed-breed livestock on farms, as was noted in a related study from mixed-breed in Northern Italy [45] [2, 13, 36]. Although a study conducted in the north-eastern areas of Colombia found that small ruminants that drink from a dam have a lower chance of being infected, our study indicates that long-standing water can get contaminated with oocytes, thus acting as a source of infection for livestock compared to boreholes [46].

## Conclusion

This study showed the seroprevalence and risk factors associated with exposure of sheep and goats to *T. gondii* infections for the first time in the North West province, South Africa. These findings further demonstrate the need for surveillance of *T. gondii* together with the rest of the reproductive pathogens in sheep and goats and should not be neglected during abortion or stillbirth cases.

### Limitation

Due to the lack of consent from the farmers in the Namusa, Lekwa-Teema, Tswaing, Ditsobotla, and Matlosana local municipalities, these municipalities were not included in the study. This was a limitation as the data obtained does not represent them.

### Electronic supplementary material

Below is the link to the electronic supplementary material.

Supplementary Material 1

## Data Availability

All data generated and analysed during this study are included in this published article.
