# Supplementary material for: Seroprevalence and risk factors of Toxoplasma gondii in sheep and goats of North West Province, South Africa
Source: BMC Vet Res. 2024 Mar 26;20:120. doi: 10.1186/s12917-024-03939-7 (PMC10964496; doi:10.1186/s12917-024-03939-7)
Supplement: Supplementary file 1 — Supplementary Material 1 [file 12917_2024_3939_MOESM1_ESM.docx]

**Additional File 1: Risk assessment questionnaire for *T. gondii* from sheep and goats in communal and commercial farms in the North West Province, South Africa**

Date:_____________

**Section A: General Information**

- 1. **Farm details:**

Province______________ District____________________ Municipality___________________

Farm/Village:________________________ GPS coordinates____________________________

- 1. **Interviewee**

| Owner | Worker | Herd man | Family | Neighbour | Other: |
| --- | --- | --- | --- | --- | --- |

- 1. **Gender**

| Male | Female |
| --- | --- |

- 1. **Age group**

| <5 | 5-18 | 18-30 | 31-39 | 40-49 | 50-59 | 60-69 | 70-79 | >80 |
| --- | --- | --- | --- | --- | --- | --- | --- | --- |

- 1. **Literacy status**

| Never went to school | Primary School | Secondary School | Tertiary School |
| --- | --- | --- | --- |

**Section B: Animal Details**

**2.1. Species**

| Sheep | Goats |
| --- | --- |

**2.2. Breed**

| **Breed** | **Number** |
| --- | --- |
|  |  |
|  |  |
|  |  |
|  |  |
|  |  |

**2.3 Sex and number of animals**

| **Sex category** | **Number** |
| --- | --- |
| Ewe |  |
| Doe |  |
| Buck |  |
| Ram |  |

**2.4 Where do you buy the animals?**

| Local market | Auction | Own bred | Other: |
| --- | --- | --- | --- |

**2.5 How long did you have the animals in the farm?**

| > 1 year | 2-5 years | >5 years |
| --- | --- | --- |

**2.6. Why do you keep them?**

| Food source | Trading | Breeding |
| --- | --- | --- |

**2.7. What type of breeding do you do?**

| Natural | Artificial insemination |
| --- | --- |

**2.8. What type of feeding system do you use?**

| Free gracing | Fed in the farm in bulk |
| --- | --- |

**2.9. How is the animal feed stored?**

**______________________________________________________________________________**

**2.10. How is the animal bedding stored?**

**______________________________________________________________________________**

**2.11. Where do the animals get the drinking water?**

| Dam | River | Borehole | Tap (Municipality) |
| --- | --- | --- | --- |

**2.12. Do you have cats present in the farm?**

| Yes | No |
| --- | --- |

**2.13. How often do you clean the animal stables/kraal?**

**______________________________________________________________________________**

**2.14. What do you do with the manure from the stables/kraals?**

**______________________________________________________________________________**

**Section C: Knowledge of Animal Reproductive Diseases Responsible for Abortions**

**3.1. Do you know that there are animal diseases that can lead to abortions in animals?**

| Yes | No |
| --- | --- |

**3.2. Do you have any animals with the history of aborting?**

| Yes | No |
| --- | --- |

**If yes, continue with question 3.3-3.5.**

**3.3. At what stage of the pregnancy does abortion occur?**

| Early | Mid | Late |
| --- | --- | --- |

**3.4. What do you do when it occurs?**

**______________________________________________________________________________**

**3.5. What do you do to the aborted foetus?**

| Burry | Burn | Leave it the stable/kraal | Feed pets | Dispose in the bin | Submit to state vet office |
| --- | --- | --- | --- | --- | --- |

**3.6. Where do you keep aborted animals?**

| Isolated from the herd | With the herd |
| --- | --- |
